# Cervical proprioception and dysphagia severity in multiple sclerosis: A cross‐sectional clinical analysis

**DOI:** 10.1111/eos.70012

**Published:** 2025-05-13

**Authors:** Hanife Abakay, M. Fatih Yetkin, Ayşe Güç, Gizem Şekercan

**Affiliations:** ^1^ Incesu Ayşe and Saffet Arslan Health Services Vocational School Kayseri University Kayseri Turkey; ^2^ Faculty of Medicine Internal Medicine Department of Neurology Erciyes University Kayseri Turkey; ^3^ Kayseri City Hospital Physical Therapy and Rehabilitation Hospital Kayseri Turkey; ^4^ Faculty of Health Sciences Department of Physiotherapy and Rehabilitation Kocaeli Health and Technology University Kocaeli Turkey

**Keywords:** dysphagia, multiple sclerosis, neck pain, proprioception

## Abstract

This study aimed to investigate the relationship between dysphagia severity and neck proprioception, pain, and mood in individuals with multiple sclerosis (MS). The individuals were divided into two groups according to the severity of dysphagia: dysphagia group (*n* = 13) with a score of 3 and above on the Dysphagia Assessment Scale in Multiple Sclerosis (DYMUS) scale and mild dysphagia group (*n* = 13) with a score of 2 and below. Clinical swallowing function was evaluated with the Turkish version of the Eating Assessment Tool‐10 (T‐EAT‐10) and DYMUS. Proprioception was assessed with a laser by noting the deviation from the center target in centimeters. Neck pain severity was assessed with the Visual Analogue Scale (VAS), and mood was assessed with the Beck Depression Inventory (BDI). Individuals with severe dysphagia demonstrated statistically significantly greater proprioceptive impairment in cervical extension and right rotation. A significant difference was also found between the two groups in terms of clinical swallowing evaluations, neck pain status, and mood measurements. The findings suggest that proprioceptive deficits, increased pain, and mood disturbances are closely associated with dysphagia severity in MS. A multidimensional approach addressing dysphagia, proprioception, and pain management may be beneficial in optimizing dysphagia rehabilitation in patients with MS.

## INTRODUCTION

Multiple sclerosis (MS) is a chronic, inflammatory neurodegenerative disorder that involves autoimmune damage to the central nervous system and is characterized by demyelinating lesions [1]. MS can lead to various clinical and neurological manifestations, including dysphagia, which refers to difficulties with swallowing [2]. A recent meta‐analysis found that dysphagia affects approximately 45% of individuals with MS [2].

Dysphagia refers to any difficulty or delay in the movement of food from the mouth to the stomach [3]. Neurogenic dysphagia is a consequence of sensorimotor impairments in the oral, pharyngeal, and esophageal stages of swallowing, typically due to neurological disorders [4]. In MS, dysphagia and its complications significantly reduce quality of life and can lead to severe outcomes, including increased morbidity and mortality in the later stages of the disease [5].

Swallowing is a complex function requiring coordinated sensory and motor activity across multiple anatomical structures [6]. The hyoid bone connects to the skull via suprahyoid muscles and to the shoulder girdle through smaller muscle groups, with indirect links to the pelvis [6]. Key structures include orofacial muscles, the mouth floor, and neck flexors [7]. Effective swallowing depends on the coordinated control of the head, neck, and trunk, with many contributing muscles located in the cervical region. In MS, dysphagia can lead to complications like malnutrition, dehydration, and aspiration pneumonia [8], underscoring the need for a holistic approach that considers the central nervous system's role in the accurate diagnosis of swallowing difficulties and the implementation of appropriate treatment strategies.

Proprioception, the sense of joint movement and positioning, is facilitated by sensory receptors [9]. The neck, where swallowing occurs, is rich in proprioceptors [10]. Alongside exteroception, proprioception aids in detecting food texture and size and maintaining head‐neck‐trunk alignment. In neurological disorders, proprioceptive dysfunction can impair body orientation [11]. Given the role of the head and neck in swallowing, proprioception plays a crucial role in maintaining postural stability and ensuring the precise coordination of muscle movements required for effective swallowing. Proprioception plays a crucial role in maintaining head and neck stability, which is essential for swallowing function [12]. Studies suggest that neurological impairments can affect proprioceptive feedback, potentially contributing to dysphagia [12]. Studies link dysphagia to proprioception deficits in neurological conditions like MS, amyotrophic lateral sclerosis, Parkinson's disease, myasthenia gravis, stroke, and ataxia [12]. While pain is common in patients with MS, it is often overlooked compared to symptoms like gait disturbances, muscle weakness, visual issues, spasticity, and bladder dysfunction [13].

The primary objective of this study was to investigate the relationship between neck proprioception and dysphagia severity in patients with MS. By integrating cervical proprioception measurements with established dysphagia evaluation tools, such as the T‐EAT‐10 and DYMUS scales, this study aimed to provide a more comprehensive understanding of the factors contributing to dysphagia in MS. The study also sought to explore whether cervical proprioceptive deficits are associated with more severe dysphagia symptoms, thereby highlighting their potential role in the pathophysiology of swallowing dysfunction in patients with MS. Ultimately, the findings of this research aim to inform future assessment methods and therapeutic approaches, offering new insights for clinical management and rehabilitation strategies for MS‐related dysphagia.

## MATERIAL AND METHODS

This single‐center clinical study was conducted between October 2022 and May 2023 at Erciyes University, Faculty of Medicine, in the Neurology outpatient clinic. Ethical approval granted by Erciyes University Non‐Interventional Clinical Research Ethics Committee with decision number 2022/692 dated 12.10.2022.

A total of 26 patients previously diagnosed with MS (20 females, 6 males; mean age 46.5 ± 7.8 years; range 30–64 years) were identified on the basis of being between 18 and 65 years of age, having a score of 24 or above on the Mini‐Mental State Examination (MMSE) test, which was required to ensure participants had intact cognitive function and to exclude those with significant cognitive impairment [14], maintaining head control, which was necessary for participation in the research. Participants who had undergone any treatment for dysphagia, had surgery involving the neck region, or had conditions affecting the cervical area, such as cervical disc herniation, radiculopathy, thoracic outlet syndrome, or rheumatic diseases like fibromyalgia, were excluded from the study. Prior to participation, individuals were provided with detailed information about the study, and those who agreed to take part signed an Informed Consent Form.

Detailed patient histories were obtained before starting the assessments. The MS type, the Expanded Disability Status Scale (EDSS) score which assesses the severity of disability in MS [15], age (years), height (cm), body weight (kg), and employment status were recorded. Afterward, clinical swallowing assessments (T‐EAT‐10, DYMUS), neck region proprioception measurements, pain status, and mood assessments were performed respectively. According to the DYMUS (Dysphagia Assessment Scale in Multiple Sclerosis) scale [16], individuals who scored 1—or 2 points were included in the mild dysphagia group, while individuals who scored between 3 and 10 points were included in the dysphagia group and divided into subgroups accordingly. All assessments in both groups were performed by a blind physiotherapist.

### Clinical swallowing assessments

#### Turkish version of the Eating Assessment Tool‐10 (T‐EAT‐10)

The Eating Assessment Tool is a valid and reliable outcome measure that includes symptom‐specific questions about the swallowing function of the person [17]. For the Turkish version, a validity and reliability study was conducted in 2016 [18]. T‐EAT‐10 is an easy and rapid dysphagia screening test consisting of 10 questions. The patient is asked to score each question between 0 and 4, from no problem (0) to severe problem (4). The scores given to each item are summed, and a total score is obtained. A high score indicates increased swallowing disorder symptom severity. A total score of 3 points or above indicates a risk for a swallowing disorder.

#### Dysphagia Rating Scale in Multiple Sclerosis (DYMUS)

Developed by Bergamaschi et al. [19], the DYMUS is a reliable and effective scale used to assess oropharyngeal dysphagia in individuals with MS. The scale consists of two subdimensions: dysphagia for solids (items 1, 3, 4, 5, 7, 8, and 10) and dysphagia for liquids (items 2, 6, and 9). Each item is scored as either “No = 0” or “Yes = 1,” and the total score ranges from 0 to 10. A “Yes” response to any item indicates the presence of dysphagia, while a score of 3 or more suggests severe dysphagia.

#### Neck region proprioception measurement

Proprioceptive sense was assessed using a laser marker placed on the patient's head [20, 21]. The assessment took place in a quiet, well‐lit environment. The patient sat comfortably in a chair with back support, and a target was positioned 90 cm away on the wall within their visual field. Prior to the assessment, the procedure and equipment were thoroughly explained.

The laser pointer device was initially aligned with the target's center by the researcher. The patient was instructed to become familiar with this position. Then, while keeping their eyes open, the patient was instructed to actively bend and focus on recognizing this position.

Subsequently, the head was flexed while the patient kept their eyes open, and they were asked to feel this position. Next, the patient was instructed to lift their head and repeat the same movement with their eyes closed, using a sleep band to block their vision. The deviation of the laser pointer from the target center was measured in centimeters, and this procedure was repeated three times. Afterward, the average of the three deviations was calculated to determine the average deviation. The same procedure was conducted for head extension and right and left rotations.

#### Evaluation of neck pain

Pain was assessed with the Visual Analogue Scale (VAS). The meanings of the numbers from 0 to 10 placed on a 10 cm line were explained. It was explained that 0 points stood for no pain, 5 points for moderate pain, and 10 points for unbearable pain [22]. Individuals were asked to tick on the line the numerical value that best expressed the level of pain they had felt in the last week.

#### Depression state assessment

The Beck Depression Inventory (BDI), developed by Beck et al. to measure the level of depression [23], to observe the differences developing as a result of treatment, and to define the disease, was used to determine the presence and degree of depression. The validity and reliability of the Turkish version of the BDI were demonstrated by Rix and Bagust in 2001 [24].

The BDI’ s validity and reliability of the Turkish version were demonstrated by Revel et al. in 1991 [20]. It can be administered individually or as a group. The severity was classified as minimal (scores 0–9), mild (scores 10–16), moderate (scores 17–29), or severe (scores 30–63).

While this sample size is adequate for the primary analysis, a total of 26 patients with MS and neurogenic dysphagia with a 5% type 1 error rate and 83% power according to the power analysis performed with the G‐Power 3.0.10 program were included in the study [12].

Data analysis was conducted using spss v.20 (SPSS). Descriptive statistics were applied, including frequency and percentage for qualitative variables, and mean, standard deviation, minimum, and maximum values for quantitative variables. The relationship between dysphagia severity and head and neck proprioception was assessed using Spearman's rank correlation test, appropriate for nonparametric data. The Mann–Whitney *U* test was employed to compare numerical variables between two independent groups under nonparametric conditions. A *p*‐value of less than 0.05 was considered statistically significant.

## RESULTS

Table 1 shows the key demographic characteristics of the participants according to their dysphagia severity. Table 1 shows the key demographic characteristics of the participants by dysphagia severity. These tests ensure that group differences in outcomes are not due to demographic factors. No significant differences were found between the groups (*p* > 0.05).

**TABLE 1:** Key characteristics of the MS participants according to the severity of dysphagia.

	Severe dysphagia (*n* = 13)		Mild dysphagia (*n* = 13)		
	Mean ± SD	Min‐max	Mean ± SD	Min‐max	*P*
**Age (year)**	47.1 ± 7.7	30–64	46.0 ± 8.2	32–57	0.959
**Height (cm)**	166.9 ± 10.1	158–195	163.2 ± 9.3	150–179	0.488
**Weight (kg)**	77.0 ± 20.9	50–125	68.4 ± 14.8	40–90	0.304
**BMI (kg/cm^2^)**	27.3 ± 4.7	19–38	25.8± 5.6	15–39	0.383
**EDSS score**	4.3 ± 1.3	2.5–6.5	2.0 ± 0.5	1.5–3.0	**0.001**
**Time since diagnosis (years)**	10.9 ± 7.0	2–20	6.9 ± 4.2	2–15	0.189

	***N* (%)**	***N* (%)**
**Gender**		
Female	10 (76.9)	10 (76.9)
Male	3 (23.1)	3 (23.1)
**MS type**		
Relapsing remitting MS	2 (15.3)	9 (69.2)
Primary progressive MS	5 (38.4)	2 (15.4)
Secondary progressive MS	6 (46.3)	2 (15.4)
**Dominant side**		
Right	12 (92.30)	11 (84.61)
Left	1 (7.70)	2 (15.39)
**Working status**		
Housewife	10 (76.9)	9 (69.2)
Working	1 (7.8)	3 (23)
Retired	2 (15.3)	1 (7.8)

Abbreviations: BMI, body mass index; EDSS, Expanded Disability Status Scale; RR, relapsing remitting; PP, primary progressive; SP, secondary progressive; Min‐max, minimum—maximum; MS, multiple sclerosis; *n*, number of participants.

Patients with severe dysphagia had higher EDSS scores (mean difference = 2.3, 95% CI = 1.5, 3.1) than patients with mild dysphagia, and they tended to have had a MS diagnosis for a longer time (mean difference = 4 years, 95% CI = ‐0.7, 8.7). In the severe dysphagia group, the most common type of MS was the secondary progressive (46.3%), whereas in the mild dysphagia group, most (69.2%) had relapsing‐remitting MS (Table 1).

Regarding proprioception measurements, a significant difference was observed between the groups in cervical extension and right rotation values (*p* < 0.05), indicating distinct differences between the groups in these specific movements. However, no significant difference was found for cervical flexion and left rotation values (*p* > 0.05), suggesting that these parameters did not vary significantly between the groups (Table 2).

**TABLE 2:** Comparison of proprioception measurement results for different head movements according to severity of dysphagia.

	Severe dysphagia(*n* = 13)		Mild dysphagia (*n* = 13)			
	Mean ± SD	Min‐max	Mean ± SD	Min‐max	Mean difference	95% CI
**Cervical flexion**	13.69 ± 7.92	5–35	9.76 ± 6.04	4–25	3.93	−1.77, 9.63
**Cervical extension**	18.61 ± 6.65	9–32	13.69 ± 10.53	3–35	4.92	−2.21, 12.05
**Right rotation**	14.61 ± 8.46	7–40	10.30 ± 6.44	5–24	4.31	−1.78, 10.40
**Left rotation**	16.07 ± 8.63	8–43	12.07 ± 5.39	7–24	4.00	−1.82, 9.82

Abbreviations: CI, Confidence interval.

Additionally, a significant difference was found between the groups in clinical swallowing evaluations, neck pain status, and mood measurements (*p* < 0.05). Both groups exhibited mild depression, with the severe dysphagia group showing a mean score of 13.92 ± 5.3, while the mild dysphagia group had a mean score of 10.0 ± 2.94 (Table 3).

**TABLE 3:** Comparison of scores for T‐EAT‐10, DYMUS, VAS, and BDI scores according to the severity of dysphagia in the sample of multiple sclerosis patients.

	Severe dysphagia (*n* = 13)		Mild dysphagia (*n* = 13)				
	X ± SD	Min‐max	X ± SD	Min‐max	Mean difference	95% CI	*p*
**T‐EAT‐10**	12.8 ± 6.78	6–27	3.00 ± 0.7	2–4	9.76	5.89, 13.65	0.001
**DYMUS**	6.28 ± 2.28	4–9	1.4 ± 0.5	1–2	4.84	3.56, 6.14	0.001
**VAS**	5.38 ± 1.68	3–8	2.8 ± 1.2	2–6	2.53	1.38–3.69	0.001
**BDI**	13.98 ± 5.4	7–25	10.0 ± 2.9	6–15	3.98	0.48‐7.49	0.05

Abbreviations: BDI, Beck Depression Inventory; CI, confidence interval; DYMUS, dysphagia in multiple sclerosis; T‐EAT‐10, Turkish version of the Eating Assessment Tool‐10; VAS, Visual Analog Scale.

The Dysphagia Rating Scale for Multiple Sclerosis (DYMUS) scores were significantly positively correlated with the pain scores (VAS), the BDI scores, and the proprioception for cervical extension, and right–left rotation values, whereas the correlation for proprioception after cervical flexion did not reach statistical significance. A significant positive correlation was also found between the T‐EAT‐10 score and the VAS and BDI scores (Table 4).

**TABLE 4:** The strength of the linear relationship between dysphagia rating (DYMUS) and swallowing function (T‐EAT‐10), respectively, and pain score (VAS), mood score (BDI), and proprioception for different head movements.

		DYMUS	T‐EAT‐10
**VAS**	**r**	0.776^b^	0.624^b^
**BDI**	**r**	0.563^b^	0.475^a^
**Cervical flexion**	**r**	0.367	0.245
**Cervical extension**	**r**	0.394^a^	0.334
**Right rotation**	**r**	0.471^*^ ^a^	0.307
**Left rotation**	**r**	0.478^a^	0.286

Abbreviations: BDI, Beck Depression Inventory; DYMUS, dysphagia in multiple sclerosis; r, correlation coefficient; T‐EAT‐10, Turkish version of the Eating Assessment Tool‐10; VAS, Visual Analog Scale.

^a^Correlation is significant at the 0.05 level (2‐tailed).

^b^Correlation is significant at the 0.01 level (2‐tailed).

## DISCUSSION

Dysphagia can be seen in patients with MS due to neurological problems and changes in muscle activation. Our findings suggest that head and neck proprioception influences dysphagia severity in MS patients. Integrating proprioceptive assessments into dysphagia management may improve swallowing function and quality of life. Our findings indicate that head and neck proprioception play a role in dysphagia severity in MS patients. Integrating proprioceptive assessments into dysphagia management could facilitate the identification of at‐risk individuals and inform targeted rehabilitation strategies to enhance swallowing function and overall quality of life. Clinical studies examining the relationship between swallowing function and the static and dynamic structures of the neck region in neurological patients and applying exercises to the neck region have been conducted [25, 26, 27]; however, there is no study in the literature showing the relationship between dysphagia severity and neck region proprioception, pain, and mood in patients with MS.

In this study, it was demonstrated that dysphagia severity is associated with impaired neck proprioception, increased neck pain, and mood disturbances in patients with MS with dysphagia at different levels. Additionally, a positive correlation was found between dysphagia severity and pain, mood, and proprioception values.

The findings of this study indicated that patients with MS and swallowing problems of different severities showed a similar distribution in terms of age, height, weight, and BMI, while dysphagia severity was associated with a higher EDSS score. Since the EDSS score reflects the overall disability level, this association suggests that greater neurological impairment may contribute to more severe swallowing dysfunction. Dysphagia is frequently observed in individuals with primary progressive and secondary progressive MS, and its risk varies according to the clinical course of MS [28]. A recent meta‐analysis evaluating the prevalence of dysphagia in MS and potential risk factors reported that dysphagia prevalence in MS is 45% and emphasized that it is a major finding that should be evaluated compared to the general population [5]. The literature suggests a relationship between EDSS score and dysphagia, indicating that dysphagia prevalence is higher in individuals with higher EDSS scores and longer disease duration [29]. The present study also showed that individuals with higher EDSS scores tend to have higher dysphagia severity, which may be explained by the involvement of neurological structures and cranial nerves at multiple levels of the central nervous system, from the cerebral cortex to the brainstem, in swallowing function [30].

As MS progresses, many motor functions are affected due to axonal loss and decreased nerve conduction velocity, leading to a decline in overall functional performance. However, MS symptoms are not limited to motor problems but also include deep and superficial sensory deficits resulting from lesions in the spinal cord, thalamus, and ascending pathways to the sensory cortex [31]. The neck region plays a crucial role in providing proprioceptive and vestibular input to the postural control system. Several studies have evaluated proprioception in different body regions to determine proprioceptive changes in patients with MS [12, 32–36]. Consistent with previous findings, the present study revealed that cervical position sense was impaired in individuals with MS and dysphagia compared to controls [36]. Given the rich proprioceptive structure of the neck region, investigating neck proprioception in this study was particularly relevant. The observation that the margin of error in proprioception measurements in patients with MS with severe or mild dysphagia exceeded the minimal threshold of 5 cm suggests a significant impairment in neck region proprioception [37].

Upon analyzing the results of neck proprioception measurements, it was observed that individuals in this study exhibited greater deviation in all measured directions. Although direct comparisons with healthy individuals were not made, these findings suggest potential impairments in proprioceptive control in MS patients. However, the group with severe dysphagia demonstrated significantly higher deviation in cervical extension and right rotation. Furthermore, a correlation was found between dysphagia severity and cervical extension as well as right and left rotation values. The absence of a statistically significant difference in proprioception measurements during flexion between the groups does not necessarily indicate the absence of a true effect. Examining the estimated differences and their confidence intervals could provide a clearer understanding of potential trends and the clinical relevance of these findings. Frequent engagement in flexion movements in daily activities may still be a contributing factor.

A 2022 study involving patients with neurological dysphagia reported significantly greater deviation in cervical flexion, extension, and left rotation in the dysphagia group [12]. The limited number of studies in this area, particularly in the context of MS, may contribute to the variability in findings across studies.

Furthermore, when evaluating the relationship between dysphagia severity and neck pain, it was noted that previous research on head and neck proprioception has primarily focused on patients with chronic neck pain, whiplash injuries, or headaches [37, 38]. These studies have reported a significant loss of proprioception in individuals with neck pain. In this study, neck pain was found to be associated with dysphagia severity, indicating that as dysphagia severity increased, neck pain intensity also increased. Given the high density of muscle spindles in the neck region, this suggests that proprioceptive input from the neck plays an important role in swallowing function.

Individuals with dysphagia often experience negative social consequences [39]. As a result, they may feel socially isolated and develop mood disturbances, including anxiety, panic, and depression [40]. A study on patients with MS reported that individuals with dysphagia experienced discomfort and increased irritability [41]. In the existing literature, studies have predominantly focused on Parkinson's disease, associating dysphagia with depression and anxiety in patients with swallowing difficulties [42, 43]. To our knowledge, this study is the first to evaluate the relationship between dysphagia severity and mood in patients with MS. The findings suggest that dysphagia severity is associated with mood disturbances, supporting the notion that dysphagia not only has physical consequences but also impacts psychological and emotional well‐being in individuals with MS.

This study has some limitations. First, the relatively small sample size may lead to increased variability in estimates, reducing precision rather than introducing bias. Potential bias would stem from the selection process and how representative our sample is of the broader MS population. Future studies should consider larger and more diverse samples to improve generalizability. Second, the cross‐sectional design allows for the identification of associations but does not establish causal relationships. While causality is not the primary focus of this study, longitudinal research would be necessary to explore whether changes in proprioception precede or result from disease progression. Additionally, our sample was limited to a specific age range and disease severity, which may not fully capture the heterogeneity of individuals with MS. Lastly, while the methods used to assess neck proprioception are widely accepted, more advanced neuroimaging or biomechanical techniques could provide further insights into proprioceptive deficits in MS.

Dysphagia is a significant health issue that adversely affects the quality of life in patients with MS, contributing to morbidity and mortality through serious complications. This study is the first to investigate the relationship between dysphagia severity and proprioception, pain, and mood in patients with MS, highlighting its unique contribution to the field. Given the high prevalence of dysphagia among patients with MS, regular monitoring is essential for early identification of dysphagia and related complications. Additionally, there is a need for well‐designed experimental studies to evaluate the efficacy of exercise programs in managing dysphagia in this patient population. Furthermore, exploring this relationship could contribute to the identification of new therapeutic targets, enhancing both diagnostic accuracy and the effectiveness of rehabilitation strategies. Such insights could ultimately improve clinical outcomes and quality of life for individuals with MS‐related dysphagia.

## AUTHOR CONTRIBUTIONS

**Conceptualization**: Hanife Abakay, Mehmet Fatih Yetkin, Ayşe Güç, and Gizem Şekercan. **Methodology**: Hanife Abakay and Gizem Şekercan. **Investigation**: Hanife Abakay, Ayşe Güç, and Gizem Şekercan. **Formal analysis**: Hanife Abakay and Gizem Şekercan. **Visualization**: Hanife Abakay, Mehmet Fatih Yetkin, Ayşe Güç, and Gizem Şekercan. **Writing—original draft**: Hanife Abakay and Gizem Şekercan. **Writing—review & editing**: Hanife Abakay, Mehmet Fatih Yetkin, Ayşe Güç, and Gizem Şekercan.

## CONFLICT OF INTEREST STATEMENT

The authors declare no conflicts of interest.

## FUNDING INFORMATION

This research received no external funding.
